# *SLC26A4* Phenotypic Variability Influences Intra- and Inter-Familial Diagnosis and Management

**DOI:** 10.3390/genes13122192

**Published:** 2022-11-23

**Authors:** Mohamed Tawalbeh, Dunia Aburizeg, Bayan O. Abu Alragheb, Wala Sami Alaqrabawi, Zain Dardas, Luma Srour, Baraah Hatem Altarayra, Ayman A. Zayed, Zaid El Omari, Bilal Azab

**Affiliations:** 1 Department of Special Surgery, Jordan University Hospital, Amman 11942, Jordan; 2 Department of Pathology and Microbiology and Forensic Medicine, School of Medicine, The University of Jordan, Amman 11942, Jordan; 3 Hearing and Speech Department, School of Rehabilitation Sciences, The University of Jordan, Amman 11942, Jordan; 4 Audiology Department, Faculty of Medical Sciences, Hacettepe University, Ankara 06100, Turkey; 5 Department of Molecular and Human Genetics, Baylor College of Medicine, Houston, TX 77030, USA; 6 King Hussein Cancer Center, Amman 11941, Jordan; 7 Division of Endocrinology, Diabetes & Metabolism, Department of Internal Medicine, Jordan University Hospital, Amman 11942, Jordan; 8 Otolaryngology, Head and Neck Surgery Department, Jordanian Royal Medical Services, Amman 11855, Jordan; 9 Department of Pathology and Cell Biology, Columbia University Irving Medical Center, New York, NY 10032, USA

**Keywords:** Pendred syndrome, enlarged vestibular aqueduct, *SLC26A4*, hearing loss, heterogeneity, DFNB4

## Abstract

*SLC26A4* is one of the most common genes causing autosomal recessive non-syndromic sensorineural hearing loss (SNHL). It has been reported to cause Pendred Syndrome (PDS) and DFNB4 which is deafness with enlarged vestibular aqueduct (EVA). However, mutated *SLC26A4* is not conclusive for having either DFNB4 or PDS. Three unrelated Jordanian families consisting of eight affected individuals with congenital bilateral hearing loss (HL) participated in this study. Whole-exome and Sanger sequencing were performed to investigate the underlying molecular etiology of HL. Further clinical investigations, including laboratory blood workup for the thyroid gland, CT scan for the temporal bone, and thyroid ultrasound were performed. Three disease-causing variants were identified in *SLC26A4* in the three families, two of which were novel. Two families had a novel pathogenic homozygous splice-site accepter variant (c.165-1G>C), while the third family had compound heterozygous pathogenic variants (c.1446G>A; p.Trp482* and c.304G>A; p.Gly102Arg). Our approach helped in redirecting the diagnosis of several affected members of three different families from non-syndromic HL to syndromic HL. Two of the affected individuals had typical PDS, one had DFNB4, while the rest had atypical PDS. Our work emphasized the intra- and inter-familial variability of *SLC26A4*-related phenotypes. In addition, we highlighted the variable phenotypic impact of *SLC26A4* on tailoring a personalized healthcare management.

## 1. Introduction

Solute carrier family 26, member 4 (*SLC26A4*; OMIM ID 605646) is a protein-coding gene for pendrin that is mainly expressed in the thyroid gland, inner ear, and kidneys [1,2,3,4]. Pathogenic variants in this gene are known to cause two autosomal recessive disorders: Pendred syndrome (PDS, OMIM: 274600) and DFNB4 (OMIM: 600791) which is deafness with enlarged vestibular aqueduct (EVA). Both phenotypes include sensorineural hearing loss (SNHL) and EVA. Mondini deformity, and vestibular abnormalities may also present in some patients with *SLC26A4*-related phenotypes [5,6]. Whereas some patients with PDS can manifest hypothyroidism with or without goiter, others may have normal thyroid function and structure [7,8,9,10]. Of note, goiter becomes apparent after the age of ten years [8]. Hypothyroidism, as one of PDS manifestations, if left untreated, can lead to different complications including cardiovascular disease, depression and infertility [10]. Therefore, it is recommended for those patients to have regular follow-up visits to optimize the L-thyroxine therapy [7].

DFNB4 has a wide spectrum of variability in terms of onset, severity, and progression. The onset of DFNB4’s HL can be triggered by head trauma [11,12,13]. EVA is considered the most penetrant feature of both PDS and DFNB4 [12]. Mixed HL and SNHL have been associated with DFNB4 [12]. Renal Pendrin plays a role in maintaining the blood’s pH, particularly in cases of metabolic alkalosis [14]. Patients with PDS should not be given thiazide diuretics as it can lead to life-threatening hypovolemia and metabolic alkalosis [15].

Only a few studies have been reported from Middle Eastern countries investigating the phenotypes associated with *SLC26A4* [16,17,18,19,20]. These studies were conducted on patients from the United Arab Emirates (UAE), Iran, and Palestine [16,17,18,19,20]. Interestingly, intra- and inter-familial variabilities were observed in some of these studies. To date, the genetic background of patients with *SLC26A4*-related phenotypes has not been reported in the Jordanian population.

In this study, we provide a detailed description of the clinical, audiological, radiological, and genetic findings of three unrelated Jordanian families initially reported with non-syndromic SNHL or mixed hearing loss (MHL). Intra- and inter-familial variability were investigated. This work highlights the gap of the absence of a conclusive correlation between *SCL26A4* variants and the associated phenotypes. We also outline the main features of the affected individuals and suggest changes for their periodic health care follow-up.

## 2. Materials and Methods

### 2.1. Participants

**Figure 1:**
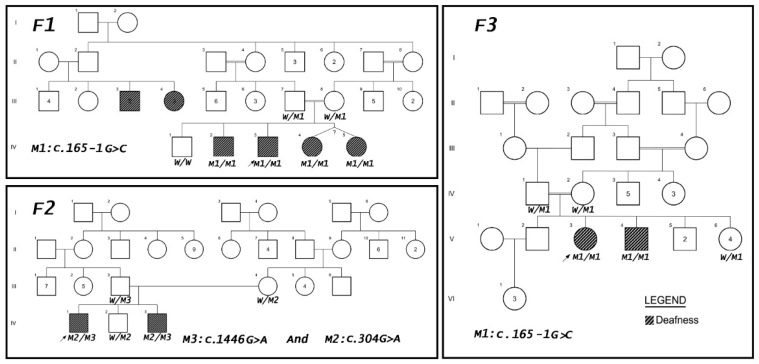
Pedigrees of the three investigated Jordanian families (F1, F2, and F3) with variants in *SLC26A4*. Females are represented by circles and males are represented by squares. Filled symbols indicate affected individuals with hearing loss while empty symbols represent unaffected individuals. Arrows point to the proband of each family. The zygosity of the identified genotypes, validated using Sanger sequencing, was presented under the symbol of affected individuals and first-degree family members. Abbreviations: W: wild type, M1: mutation c.165-1G>C, M2: mutation (c.304G>A; p. Gly102Arg), and M3: mutation (c.1446G>A; p.Trp482*).

Seventeen members from three unrelated Jordanian families were recruited in this study, eight of which were initially diagnosed with non-syndromic SNHL or MHL (Figure 1). Written informed consents were taken from all participating members including the children’s guardians. All research was conducted in conformity with the defined ethical standards of the declaration of Helsinki and was approved by the institutional review board (IRB) (IRB number 2016/110) of Jordan University Hospital (JUH).

### 2.2. DNA Extraction

Peripheral blood samples were withdrawn from all participating members, and a detailed clinical history was taken prior to evaluation. gDNA was obtained from whole blood using Promega Wizard Genomic DNA Purification Kit (Promega, Madison, WI, USA) following the instructions provided by the manufacturer. DNA was stored at −20 °C after examining its purity and concentration using NanoDrop 2000 spectrophotometer (Thermo Fisher Scientific, Waltham, MA, USA).

### 2.3. Whole-Exome Sequencing and Data Analysis

Whole-exome sequencing (WES) was performed for the proband of each family (F1: IV-3, F2: IV-1, and F3: V3), as described elsewhere [21]. Our filtration approach was based on gene list-focused analysis (Table S1). The gene list was generated by compiling the genes from deafness-related literature, OMIM (https://omim.org; last accessed 19 November 2022), Hereditary Hearing Loss Homepage (HHL; https://hereditaryhearingloss.org; last accessed 19 November 2022), and Deafness Variation Database (DVD; https://deafnessvariationdatabase.org; last accessed 19 November 2022). The variants were prioritized according to the following steps: (a) variants located in the coding and flanking region; (b) variants with minor allele frequency (MAF) for subpopulations of ≤1% in gnomAD (https://gnomad.broadinstitute.org; last accessed 19 November 2022), TOPMed (https://topmed.nhlbi.nih.gov; last accessed 19 November 2022), 1000 Genomes Project (http://www.internationalgenome.org; last accessed 19 November 2022), NHLBI Exome Sequencing Project (https://evs.gs.washington.edu/EVS/; last accessed 19 November 2022), and DGV (http://dgv.tcag.ca/dgv/app/home; last accessed 19 November 2022); (c) total read depth of ≥10× reads. We compiled a list of the variants that fulfilled the previous filtration approach (Table S2). The short variant list underwent further analysis based on the mode of recessive inheritance, patient clinical picture, relevant literature, and available data from HGMD (http://www.hgmd.cf.ac.uk/ac/index.php; last accessed 19 November 2022), OMIM (https://omim.org; last accessed 19 November 2022), GeneCards (www.genecards.com; last accessed 19 November 2022), and varsome (https://varsome.com; last accessed 19 November 2022). Variant classification was based on expert specifications of the ACMG/AMP guidelines for genetic hearing loss [22].

### 2.4. Co-Segregation Analysis

Primers flanking the identified candidate variants were designed. Sequences of the forward and the reverse primers and their PCR conditions are presented in Table S3. Co-segregation of the disease with the identified candidate variants was performed by bi-directional Sanger sequencing. The diagrammatic representation of *SLC26A4* was created using Lollipops variant visualization tool (https://joiningdata.com/lollipops/index.html; last accessed 19 November 2022).

### 2.5. Clinical Examination

Individuals had a detailed clinical evaluation. Pure tone audiometry was performed for the affected patients at the time of the study. Previous history of audiological assessments was retrieved from the medical records of the patients. High-resolution temporal bone computerized tomography (CT) scan without contrast was performed for all affected individuals to look for EVA. EVA is considered enlarged if its midpoint width is more than or equals to 1.5 mm based on Valvassori and Clemis criterion. Thyroid ultrasound (U/S) and thyroid function tests including serum thyroid stimulating hormone (TSH), serum free thyroxine (FT4), serum free triiodothyronine (FT3), anti-thyroid peroxidase antibodies (anti-TPO Ab), thyroglobulin (Tg), and anti-thyroglobulin antibodies (anti-Tg Ab) were evaluated for affected members. Previous medical records were obtained and investigated thoroughly.

## 3. Results

### 3.1. Audiological Findings

Participants from three unrelated Jordanian families presenting with the initial diagnosis of non-syndromic SNHL or MHL and their unaffected first-degree family members were included in this study for genetic assessment (Figure 1).

**Figure 2:**
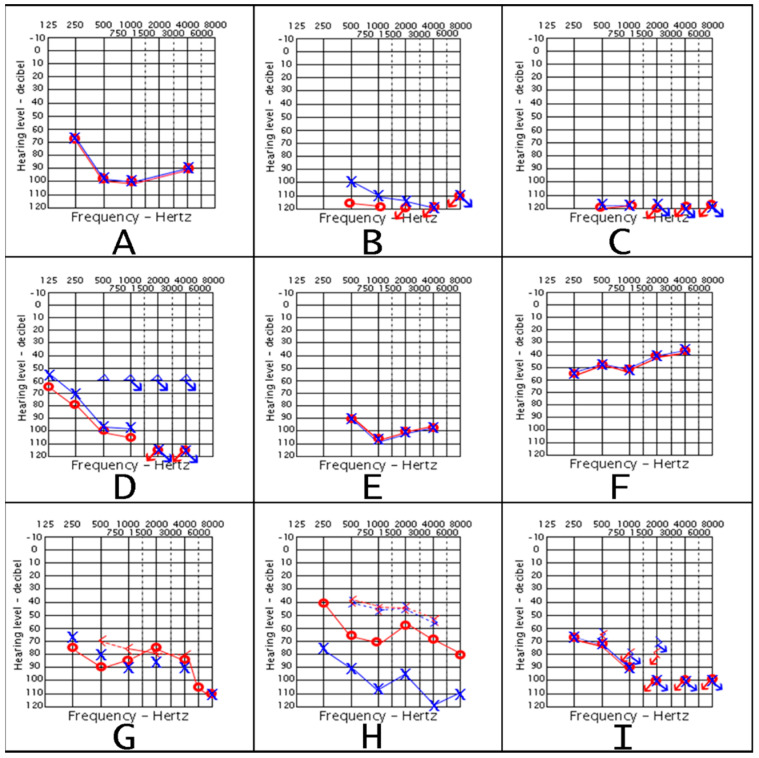
Audiograms of selected family members. Audiograms of F1: IV3 (c.165−1G>C) are shown in (**A**–**C**) at the age of 1 year and 7 months, 6 years, and 17 years, respectively. (**D**) for F1: IV-2 at the age of 3 years. Audiograms of F1: IV−5 are depicted in (**E**,**F**) before and after cochlear implant at the age of 4 and 5 years, respectively. (**G**) for F2 (p.Gly102Arg and p.Trp482*): IV−1 at the age of 7 years. (**H**) for F2: IV-3 at the age of 5 years. (**I**) for F3 (c.165−1G>C): V−4 at the age of 20 years. The symbols of the red circles and the blue crosses represent the readings of earphones unmasked air conduction of the right and left ear, respectively. Blue triangles for the masked air conduction readings of the right ear. The (<) and (>) symbols are used to represent the reading of the mastoid unmasked air conduction of the right and left ears, respectively. Arrows on any of the symbols represent no response.

We examined the audiological and hearing loss status of the affected members of the three families (Table 1 and Figure 2). F1 had four affected siblings (IV−2, IV−3, and the twins IV−4, and IV−5) and they all had congenital severe to profound SNHL that is progressive and bilateral (Figure 2A–D). The elder brothers (IV−2; 29 years and IV−3; 25 years) who had moderately severe to profound SNHL are completely deaf now as they did not receive any HL intervention. Figure 2A–C shows the progression pattern of one of the elder brothers (IV−3) which progressed from severe sloping at the age of 1 year and 7 months to flat profound at 6 years to dead ears at 17 years (Figure 2A–C). However, the twins (IV−4 and IV−5; 17 years) had unilateral cochlear implant at the right side at the age of 9 years which improved their HL from profound to moderate (Figure 2E,F). Speech rehabilitation for the twins continued until high school age. Moreover, distant family members were reported to have HL.

F2 had two affected young siblings (IV−1; 9 years and IV−3; 5 years). They had SNHL and MHL, and the onset was at 2 years. Their HL has been deteriorating constantly and the eldest affected child, IV−1, had more severe HL in comparison to his younger sibling IV−3. Both affected siblings (IV−1 and IV−3) are currently using hearing aids (Figure 2G,H).

F3 had two affected siblings (V−3; 16 years, and V−4; 23 years). V−4 had severe to profound progressive MHL that started at the age of 1 year and 4 months while V−3 had moderately severe SNHL that has started at the age of 3.5 months (Figure 2I). They are both currently using hearing aids.

### 3.2. WES Findings 

The proband of each family (F1: IV−3, F2: IV−1, and F3: V3) underwent WES which identified three disease-causing variants (DCV) in the *SLC26A4* gene (Transcript ID: NM_000441.1, Table 2).

#### 3.2.1. Family F1 and Family F3 WES Findings

A novel homozygous splice-site accepter variant (c.165−1G>C) in *SLC26A4* was identified in F1 and F3. In silico variant assessment tools predicted this splice variant to cause a splice site loss (Table 2). This variant is absent from the population database (gnomAD; https://gnomad.broadinstitute.org; last accessed 19 November 2022) and is not listed in ClinVar. Loss of function (LoF) variants are a known mechanism of pathogenicity in *SLC26A4*, and LoF variants have been previously reported as disease-causing near the same splicing region [5,23]. Furthermore, this variant co-segregated with the disease in both families. Collectively, we classify this LoF variant as pathogenic.

#### 3.2.2. Family F2 WES Findings

We found two variants in *SLC26A4* (c.1446G>A and c.304G>A). The first variant (c.1446G>A; p.Trp482*) is a novel nonsense variant in exon 13. Nonsense-mediated mRNA decay (NMD) is predicted to occur as the distance between this stop codon and the nearest 3’ end of the exon–exon junction is >55 nucleotides long. This LoF variant was not available in either ClinVar or gnomAD databases. Several studies reported pathogenic LoF variant in exon 13 [24,25].

The second variant in F2 is a missenses variant (c.304G>A; p.Gly102Arg) and is located in the splicing region of exon 3. This change introduced arginine instead of glycine at residue number 102. This variant was reported in ClinVar (ID: VCV001301849, classified as pathogenic/likely pathogenic; last accessed 24 April 2022) and gnomAD (total allele frequency: 0.000003978). This variant has been previously reported in Iranian patients affected with SNHL and with PDS [26,27,28,29]. Further, co-segregation analysis for F2 revealed these two variants (c.1446G>A and c.304G>A; Table 2 and Figure 1) to be in *trans*. Given together, we classified these two variants as pathogenic.

### 3.3. Co-Segregation Analysis

**Figure 3:**
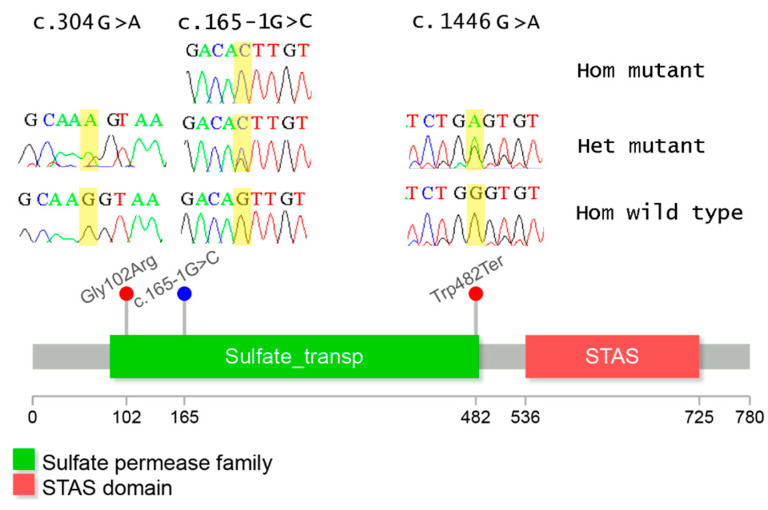
Schematic representation of the location of the variants in SLC26A6. The upper pane represents the corresponding chromatograms of the identified variants. Variant locus is highlighted in yellow. Black peak, G base; blue peak, C base; red peak, T base; green peak, A base. Abbreviations: Hom: homozygous, Het: Heterozygous.

The three candidate variants were further investigated for co-segregation by Sanger sequencing (Figure 1 and Figure 3). All affected individuals in F1 (IV-2, IV-3, IV-4, and IV-5) and F3 (V-3 and V-4) were homozygous for the sequence change (c.165-1G>C). The parents (F1: III-7 and III-8, F3: IV-1 and IV-2) were heterozygous carriers. The available unaffected sibling was either heterozygous (F3: V-6) or homozygous for the wild type allele (F1: IV-1). Regarding F2, the unaffected father harbored the nonsense variant (c.1446G>A; p.Trp482*) in a heterozygous state, while the mother had the missense variant (c.304G>A; p.Gly102Arg) in a heterozygous state. The affected F2 members (IV-1 and IV-3) were found to be bi-allelic for c.1446G>A and c.304G>A. Their unaffected sibling (IV-2) was heterozygous for c.304G>A (Figure 1 and Figure 3).

Pathogenic variants in *SLC26A4* are associated with DFNB4 (OMIM: #600791) and PDS (OMIM: #274600). These genetic findings urged us to further investigate inner ear malformations that are associated with DFNB4, and thyroid manifestations that might be due to PDS in affected individuals.

### 3.4. Temporal Bone CT Findings

**Figure 4:**
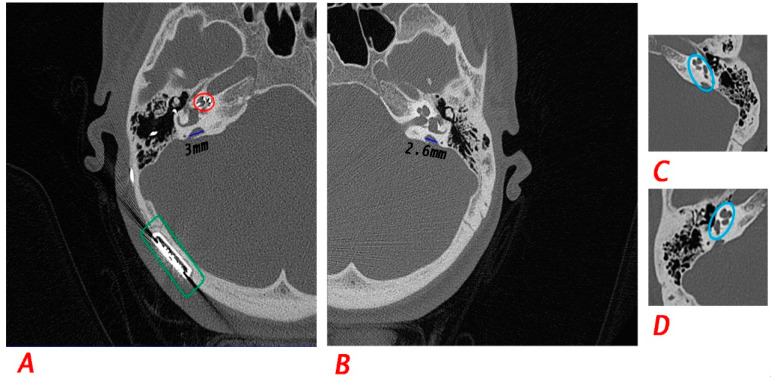
Temporal CT scan findings for F1: IV-3 and F1: IV-4. (**A**,**B**) Axial view of bilateral temporal bone CT scan for F1: IV-4 at the age of 17 years old post cochlear implantation where the blue lines represent the length of the EVA. The red circle shows the electrodes of the cochlear implant and the green rectangle shows the implanted cochlear device (**A**) for the right side and (**B**) for the left side. (**B**,**C**) Axial view of bilateral temporal bone CT scan for F1: IV-3 at the age of 25 years old showing features of incomplete partition II (Mondini Deformity) indicated by the blue circle (**C**) for the left side and (**D**) for the right side.

Affected members of the three families were examined by temporal CT for inner ear malformations (Table 1 and Figure 4). Temporal CT scan of the four affected siblings in F1 (IV−2, IV−3, and the twins IV−4, and IV−5) showed that they had bilateral asymmetrical EVA. Moreover, the two brothers (IV−2 and IV−3) had bilateral focal dehiscence of the posterior semi-circular canals. IV−3 had appearances suggestive of Mondini deformity as well. IV−3’s CT scan showed unremarkable right external auditory canal, clear mastoid air cells, antrum and middle ear cleft, and normal morphology and alignment of the middle ear ossicles. The basal cochlear turn of IV−3 appears normal. The cochlear apex appears cystic due to fused middle and apical turns. The dilated vestibular aqueduct and significant thinning of the bony roof of the posterior semicircular canal suggestive of dehiscence were found in IV−3 CT scan as well. Normal appearances of the internal auditory canal, carotid canal, jugular bulb and cochlear aqueduct. Normal appearances of the lateral and superior semicircular canals were detected. The twins (IV−4 and IV−5; 17 years) were the only affected members to experience dizziness and vertigo.

F2 affected siblings (IV−1 and IV−3) showed bilateral asymmetrical EVA for both of them and focal bony dehiscence of the posterior semicircular canals for IV−3 bilaterally. No dizziness or vertigo was reported. V−3 and V−4 from F3 denied having vertigo and dizziness. CT scan of F3: V−4 showed bilateral asymmetrical EVA. Given together, all of the diagnostic criteria of DFNB4 associated with *SLC26A4* were met in the three families with some variability. All affected members had early-onset (between 0 to 2 years) bilateral progressive SNHL or MHL. The severity of the HL ranged from moderate to profound and it was associated with vertigo or dizziness in only the twins (F1: IV−4 and IV−5). Available CT scans of all affected individuals revealed that they have bilateral and asymmetric EVA at variable widths exceeding 1.5 mm. Three of the affected individuals (F1: IV−2 and IV−3, F2: IV−3) have shown to have focal dehiscence of the posterior semi-circular canals bilaterally. Moreover, only one patient (F1: IV-3) had appearances suggestive of Mondini deformity. The MHL of IV−3 can be due to chronic otitis media with effusion which indicated medical treatment and grommet tube insertion. At the time of examination, the tubes were no longer present in the ear, but the surgical site was complicated with perforation on the right side and glue was still present on the left side. There was an incomplete closure of air-bone gap on the right side and partial incomplete closure on the left side. Moreover, the MHL can also be due to the CT findings of the focal bony dehiscence of the posterior part of the superior semicircular canals bilaterally (Figure 4). The genetic testing pinpointed the two-differential diagnoses for the affected individuals. However, thyroid endocrine profiling would settle on their diagnosis as DFNB4 or PDS.

### 3.5. Thyroid Findings

We examined the clinical features suggestive of PDS for the three affected families (Table 3). F1 affected siblings (IV−2, IV−3, and the twins IV−4, and IV−5) showed wide variability in their findings. The eldest two siblings (IV−2 and IV−3) have normal sized thyroid gland without lesions in their thyroid U/S. Their blood tests were all normal except for Tg which was only elevated in IV-3. Thyroid U/S for the twins (IV−4 and IV−5) showed diffuse goiter with multiple bilateral small anechoic oval nodules and heterogeneous echogenicity. It is worth mentioning that F1: IV-4 was diagnosed with hypothyroidism four years ago and required L-Thyroxine therapy; however, she has not been compliant with it. The blood tests of her twin (IV-5) showed that she was euthyroid. Although Tg was elevated in both, it was 10−fold higher in IV−4.

Both of F2 affected members were similar in findings of the affected individual F1: IV−3. Thyroid gland size was normal by U/S without any nodularity. All blood tests were within the normal range except for Tg which was elevated in both patients. We have tested their unaffected young brother F2: IV−2 as a control to make sure if his Tg level was also elevated, which apparently was within the normal range.

F3 affected members (V−3 and V−4) did not have any goiter. Thyroid U/S of F3: IV−4 was normal in size and echogenicity.

The genetic findings of *SLC26A4* along with the clinical examination and investigations suggested a variable set of differential diagnosis for the affected patients. Variability was noted in inter- and intra-familial levels.

## 4. Discussion

In this study, we performed genetic and clinical analyses on three unrelated Jordanian families with SNHL or MHL (Figure 1). After WES analysis, we identified three variants in *SLC26A4*, two of which (c.165−1G>C and p.Trp482*) were novel (Table 2). This is the first report for patients with PDS-suggestive findings to be molecularly investigated in Jordan.

*Genetic Findings in SLC26A4.* All of the affected individuals from the three investigated families had bi-allelic alterations in *SLC26A4.* Members with HL from F1 and F3 were homozygous for the same splice-site variant (c.165−1G>C), while those in F2 had compound heterozygous variants (p.Trp482* and p.Gly102Arg; Figure 1 and Figure 3). However, variants in *SLC26A4* causing DFNB4 or PDS can also be di-genic with other genes such as *FOX1, EPHA2,* and *KCNJ10* [30,31,32,33,34]. These genes did not harbor any candidate DCVs in our cases. This implies that the described variabilities in the *SLC26A4*-related phenotypes in our families are not driven by di-genic variants in the aforementioned genes. 

*Audiological findings in the context of the DCVs.* Previous studies correlated the number of mutated alleles in *SLC26A4* to the severity of DFNB4. Bi-allelic variants led to higher inner ear fluid pressure, worse hearing threshold, and more severe inner ear malformations by having a wider EVA and Mondini defect [13]. All the patients in this study had early-onset SNHL that is pre-or peri-lingual, progressive, associated with EVA, and sometimes vestibular abnormalities. These described audiological findings were compatible with the differential diagnosis of DFNB4 and PDS. Cochlear implants for *SLC26A4*-related cases demonstrated better residual hearing than those who did not [11]. Likewise, in our study, audiograms of one of the twins (F1: IV−5) before and after the cochlear implantation showed improvement. This can be noticed by comparing the audiograms of her two elder siblings (F1: IV−2 and IV−3) who did not have a cochlear implant and are completely deaf now (Figure 2 and Table 1) [11]. All cases affected by *SLC26A4*-related phenotypes require periodic audiological examinations to calibrate their hearing aids [8]. Cochlear implants are advised to be used to prevent these patients from being mute.

*Thyroid findings in the context of the DCVs.* Since DFNB4 and PDS have multiple overlapping features, thyroid manifestations are considered the distinguishing feature between *SLC26A4*’s associated phenotypes [7]. Typically, PDS is associated with goiter and accompanying overt hypothyroidism, subclinical hypothyroidism, or euthyroidism [7]. Goiter in PDS usually develops in late childhood and early adolescence, making it difficult to diagnose PDS at a young age [35]. To evaluate thyroid function, several investigations were carried out, including thyroid U/S and other lab testing [7].

*Families with the homozygous splice-site variant (c.165−1G>C)*. Thyroid investigations for the four affected individuals from F1 who had the splicing variant (c.165−1G>C) showed that only one patient (F1: IV−4) had hypothyroidism with goiter, which is typical for PDS. However, the rest of the four affected members were euthyroid. The only euthyroid patient with diffuse enlargement and nodularity in thyroid U/S was F1: IV-5 (the twin of F1: IV−4). Thus, she was also diagnosed with PDS. Similarly, Sakurai et al. showed that identical twins with PDS can have different levels of progression [36]. Particularly, although both had thyroid nodules, one of the twins developed papillary thyroid carcinoma whereas the other twin’s nodules were benign [36]. Several studies showed that PDS nodules can progress to thyroid carcinoma [36]. This association was validated functionally by low *SLC26A4*’s mRNA expression and low immunostaining for pendrin in cancerous thyroid tumors [36,37]. Therefore, we recommend periodic follow-up investigations for the investigated twins (F1: IV−4 and F1: IV−5) to exclude any possible malignant transformation in the future.

The two other affected siblings in this family (F1: IV−2 and IV−3) had different symptoms than the twins (F1: IV−4 and IV−5). The individuals (F1: IV−2 and IV−3) did not have goiter, favoring the diagnosis of DFNB4 rather than PDS. Nevertheless, the elevated Tg in F1: IV−3 triggered the suspicion of atypical PDS [7,35]. Elevated serum Tg is a non-specific finding of PDS and can be associated with different thyroid pathologies [38]. In compliance, elevated Tg levels have been reported in PDS patients who do not have goiter [35]. Therefore, F1: IV−3 should have periodic thyroid function tests to monitor the potential progression of thyroid disorder.

F3 had the same DCV that was identified in F1 (c.165−1G>C). Available findings for F3: V−4 (MHL, EVA, and normal thyroid U/S) are suggestive of DFNB4. However, this cannot exclude the possibility of PDS as further thyroid investigations are needed. Therefore, further surveillance is needed to diagnose any delayed thyroid abnormalities.

In the same splicing region of the identified variant in F1 and F3 (c.165−1G>C), another pathogenic variant was reported by Trevino et al. (c.165−1G>A) [23]. The previously reported variant (c.165−1G>A) was compound heterozygous with another likely pathogenic missense variant (p.Ala411Pro). The siblings, harboring the variant (c.165−1G>A), had PDS. The elder sibling had a significantly enlarged goiter at the age of 20 years, but she was clinically euthyroid. This sibling had prelingual SNHL diagnosed at the age of 6 months. Her clinical picture was similar to that of F1: IV−5, who had congenital SNHL and euthyroid nodularity except that her goiter was not as large as the elder sibling’s as reported by Trevino et al [23]. This could be a sign that the nodules in F1: IV−5 might enlarge over time, resulting in large neck goiter in their twenties. This necessitates close monitoring of F1: IV−5 by endocrinologists. The younger sibling reported by Trevino et al [23]. had congenital HL and was mute. He had delayed motor milestones and congenital hypothyroidism. His large goiter was noted at 9 years of age. Although he was treated with iodine, he presented clinically with hypothyroidism and mental retardation at the age of 14 years [23]. Compared with F1: IV−4, he had a more severe and earlier onset type of PDS.

Furthermore, the siblings reported by Trevino et al. [23] had Mondini deformity, which was not found in the two PDS patients in F1 (IV−4 and IV−5). Only F1: IV−3 had the Mondini deformity, which we considered in his diagnosis as atypical PDS with elevated Tg and congenital SNHL. Although these two splicing variants (c.165−1G>A and c.165−1G>C) are located in the same position, inter- and intra-familial phenotypic variability can be observed. This can be attributable to yet-to-be-identified modifier genes.

*A family with compound heterozygous variants.* The two affected children in F2, IV−1 and IV−3, had compound heterozygous variants (p.Trp482* and p.Gly102Arg). Thyroid investigations for these two children showed elevated Tg levels and euthyroid without goiter. Their normal thyroid size can be attributed to their young age [35]. Their third unaffected young brother (F2: IV-2) had normal Tg levels. Based on these tests, we diagnosed F2: IV−1 and IV−3 as atypical PDS. A member from F1, IV−3, also had atypical PDS, albeit with different variants in *SLC26A4*. Their management plan should include a multidisciplinary approach by pediatric, endocrine, and ear, nose, and throat (ENT) physicians. Moreover, their parents should be informed that they have normal thyroid function tests but that they require regular follow-ups. Cochlear implants should be initiated for them as soon as possible.

One of the variants in F3 (p.Trp482*) has not been reported before, while the other (p.Gly102Arg) has been reported in several studies [26,27,28,29]. Taylor et al. reported the variant (p.Gly102Arg) in a homozygous state in a PDS patient presenting with goiter and hypothyroidism [26]. He was diagnosed with HL at 17 months [26]. In our study, affected members in F2 had a comparable age of HL onset at 24 months. In addition, Cengiz et al. reported the same variant with SNHL without any indications of thyroid or EVA [28]. A functional study on this variant by minigene splicing assay (p.Gly102Arg) detected abnormally spliced transcripts that resulted in altered pendrin function and loss of iodide efflux [27,29].

The nutritional status of iodine affects the onset of thyroidal abnormalities and the development of goiter in PDS patients [7]. We do not think that it had an effect on our patients as they are all living in the same region in Jordan where we have adequate iodine levels with a urine iodine concentration median of around 203 μg/L [39]. Moreover, unaffected family members and their family history are not known to have any thyroid manifestations.

This study showed a wide variety of phenotypes associated with *SLC26A4* variants at inter- and intra-familial levels. Only two patients had PDS (F1: IV−4 and F1: IV−5), one had DFNB4 (F1: IV−2), and the remaining patients had atypical PDS (F1: IV-3 and F2: IV-1 and IV−3). Unfortunately, we could not formulate a deferential diagnosis for F3: V−2 and V−4 due to the limited available data.

*SLC26A4 in the Middle Eastern countries.* Two families from the United Arab Emirates were reported to have disease-causing variants in *SLC26A4* [16]. One of them had findings suggestive of PDS within all affected family members, while the other had DFNB4 [16]. Another study conducted in Palestine revealed two families with variants in *SLC26A4* [17]. One of these families was reported to have members with either DFNB4 or atypical PDS; the individuals from the other family were found to have severe to profound HL [17]. Moreover, three studies were reported from the Iranian population and described variable inter- and intra-familial manifestations in the *SLC26A4*-related phenotypes [18,19,20]. Collectively, these findings highlight that inter-and intra-familial variability of *SLC26A4-*related phenotypes in the Middle East region can be evident in reports of certain countries but not others.

Previously, no significant associations were drawn between the types of *SLC26A4* variants, the onset of HL, and the tendency for progression [13,40,41,42,43,44,45,46]. However, PDS patients have been marked to have a worsening outcome of HL compared to DFNB4 patients. We believe this supports the idea that DFNB4 and PDS are continua to each other [13]. A study on several *SLC26A4* variants causing either PDS or DFNB4 showed that pendrin function, which is iodide and chloride transportation to thyrocytes, was completely lost in PDS but some of these ions were transported in DFNB4 [47]. This suggested that the minimal residual function of pendrin in DFNB4 is good enough to prevent or delay the onset of goiter [47]. Moreover, this wide variability in clinical aspects suggests that there are other modifier genes or epigenetic changes affecting the phenotypes.

Given the variable phenotypic impact of *SLC26A4,* healthcare management needs to be personalized. Typical PDS patients should follow up for malignant transformation. Thiazide diuretics should not be administered to PDS patients to avoid hypovolemia and metabolic alkalosis [14,15]. Atypical PDS patients, even if they do not manifest any thyroid-related symptoms, should have the thyroid profile added to their periodic healthcare plan. DFNB4 patients, along with typical and atypical PDS, should be observed by ENT physicians. 

## 5. Conclusions

This study further stresses the variability associated with mutated *SLC26A4*’s phenotypes and reports them for the first time in the Jordanian population. We identified three variants in *SLC26A4*, two of which were novel. We studied the associated phenotypes, which were variable at the intra-and inter-familial levels. Augmenting our current findings with the previous and future corpus of genetic findings might pave the way to the establishment of genotype–phenotype correlations. This study reveals the importance of tailoring personalized medical care for individuals with variants in *SLC26A4* based on their associated phenotype. Early diagnosis of *SLC26A4*-related phenotypes can improve the management of potential thyroid manifestations. This work creates an avenue towards considering *SLC26A4* as part of the molecular testing for children presenting with congenital or early-onset SNHL.

## Figures and Tables

**Table 1:** Audiological findings and investigations of the affected members.

F#	Individual	Age	Sex	Age at HL Onset	Severity of HL		HL Progression	Vertigo/Dizziness	SNHL Intervention	High-Resolution Temporal Bone Computerized Tomography (CT) Scan without Contrast
					Right Ear	Left Ear				
1	IV−2	29	M	Congenital	Severe to profound SNHL	Moderately severe to profound SNHL	Yes	No	Completely deaf	Enlarged vestibular aqueducts (Rt 1.5 mm, Lt 2 mm). Focal dehiscence of the posterior semi-circular canals bilaterally. Prominent/Slightly dilated vestibule.
	IV−3	25	M	Congenital	Profound SNHL	Profound SNHL	Yes	No	Completely deaf	Appearances are suggestive of Type II incomplete partition (Mondini deformity) bilaterally manifested by fused cystic cochlear apex as well as enlarged vestibular aqueducts. (Rt 2 mm, Lt 2.5 mm). Focal dehiscence of the posterior semicircular canals bilaterally.
	IV−4	17	F	Congenital	Profound SNHL	Profound SNHL	Yes	Yes	Cochlear implant at 9 years old	Both vestibular aqueducts are dilated (Rt 3 mm, Lt 2.6 mm).
	IV−5	17	F	Congenital	Profound SNHL	Profound SNHL	Yes	Yes	Cochlear implant at 9 years old	Both vestibular aqueducts are dilated (Rt 2 mm, Lt 2.2 mm).
2	IV−1	9	M	2 years	Severe to profound SNHL	Severe to profound SNHL	Yes	No	Hearing aids	Enlarged vestibular aqueducts (Rt 3.5 mm, Lt 3 mm). Mildly dilated vestibule bilaterally.
	IV−3	5	M	2 years	Moderate to severe MHL	Severe to profound MHL	Yes	No	Hearing aids	Enlarged vestibular aqueducts (Rt 2.4 mm, Lt 1.7 mm). Mildly dilated vestibule bilaterally. Probable focal bony dehiscence of the posterior part of the superior semicircular canals bilaterally. These also showed slightly small lumen and faint sclerosis compared to the anterior part of the semicircular canal.
3	V−3	16	F	3.5 months	Moderately severe SNHL	Moderately severe SNHL	Yes	No	Hearing aids	Not available.
	V−4	23	M	1.3 years	Severe to profound SNHL	Severe to profound SNHL	Yes	No	Hearing aids	Bilateral enlarged vestibular aqueduct (Rt 1.9 mm, Lt 3 mm).

Abbreviations: SNHL: sensorineural hearing loss, MHL: mixed hearing loss, Rt: right, Lt: left.

**Table 2:** Candidate variants identified by whole-exome sequencing in the three families.

Family Number	Gene	Variant Coordinate		Exon	HGVS cDNA	HGVS Amino Acids	Transcript	Consequences	ClinVar, Last Accessed 12 July 2022	Maximum Minor Allele Frequency gnomAD	Zygosity	In-Silico Predictions	ACMG Classification	Reference
		hg38	hg19											
1 and 3	*SLC26A4*	chr7:107663295	chr7:107303740	-	c.165−1G>C	-	NM_000441.1	Splice acceptor	Not reported	V2/V3: Absent	Homozygous	- NetGene2: Not detected acceptor site - NNSplice: Not detected acceptor site - MaxEntScan: High impact - SpliceAI: Acceptor loss	Pathogenic	
2		chr7:107663435	chr7:107303880	3/21	c.304G>A	p.Gly102Arg		Missense Splice region	Reported (Variation ID:1301849)	V2: 0.000008800 V3: Absent	Compound Heterozygous	REVEL score: 0.944 (Pathogenic) SIFT: 0 (Damaging) PROVEAN: 0.9569 (Damaging)	Pathogenic	[26,27,28,29]
		chr7:107695941	chr7:107336386	13/21	c.1446G>A	p.Trp482*		Nonsense	Not reported	V2/V3: Absent		Nonsense-mediated decay is predicted	Pathogenic	

Abbreviations: ACMG, American College of Medical Genetics; gnomAD, the genome aggregation database; HGVS, human genome variation society; V, version.

**Table 3:** Clinical characteristics and investigations of the thyroid gland.

F#	Individual	Age	Visible Goiter	FT3 (1.2–4.1 pg/mL)	FT4 (0.89–1.72 ng/dL)	TSH (0.4–4.5 mIU/mL)	TG (3.5–77 ng/mL)	Anti-TG Ab (Up to 115.0 IU/mL)	Anti-TPO Ab (Up to 30 IU/mL)	Thyroid Ultrasound
1	IV−2	29	No	3.4	1	2	66.08	<10.0	3.2	Normal size, echo pattern, and blood flow of the thyroid gland. No thyroid nodules are seen. Normal submandibular glands. No cervical lymph node enlargement. Right thyroid lobe measured about 1.5 × 1.6 × 4.6 cm. Left thyroid lobe measured about 1.7 × 1.1 × 5 cm.
	IV−3	25	No	3.2	1.1	4	112	<10.0	1.3	Normal size, echo pattern, and blood flow of the thyroid gland. No thyroid nodules are seen. Normal submandibular glands. No cervical lymph node enlargement. Right thyroid lobe measured about 2.1 × 1.6 × 4.8 cm. Left thyroid lobe measured about 1.8 × 1.3 × 5.1 cm.
	IV−4	17	Yes	3.8	0.869	5.27	1000	Negative	Negative	Both thyroid lobes and isthmus are diffusely enlarged. Isthmus AP diameter of 8.9 mm. Multiple bilateral anechoic oval nodules some with septation. Heterogeneous course echo texture.
	IV−5	17	No	3.7	1.08	2.23	138	Negative	Negative	Both thyroid lobes and isthmus are enlarged. Isthmus AP diameter 5 mm. Multiple bilateral small anechoic nodules. There is well defined hypo echoic and anechoic nodule of 9 × 3.5 mm in the left lobe close to the isthmus. Heterogeneous course echo texture.
2	IV−1	9	No	2.4	1.4	1.2	130.6	10.6	8.7	Normal size, echo pattern, and blood flow of the thyroid gland. No thyroid nodules are seen. Normal submandibular glands. No cervical lymph node enlargement. Right thyroid lobe measured about 1.7 × 1.7 × 4.2 cm. Left thyroid lobe measured about 1.2 × 1.7 × 4 cm.
	IV−3	5	No	3	1.3	1.6	96.59	11.8	4.9	Normal size, echo pattern, and blood flow of the thyroid gland. No thyroid nodules are seen. Normal submandibular glands. No cervical lymph node enlargement. Right thyroid lobe measured about 1 × 1.5 × 3.6 cm. Left thyroid lobe measured about 1.5 × 1.4 × 3.1 cm.
	IV−8	7	No	3.8	1.57	1.4	18.1	11.8	4.7	-
3	V−3	16	No	-	-	-	-	-	-	-
	V−4	23	No	-	-	2.02	-	-	-	Both thyroid lobes and isthmus appear normal in size and echogenicity. The right thyroid lobe measuring 2.5 × 3.7 × 4.6 cm. The left thyroid lobe measuring 2.7 × 2.5 × 5.7 cm. No cystic or solid mass lesions could be seen. No significant enlarged lymph nodes could be seen on both sides of the neck.

Abbreviations: U/S: Thyroid ultrasound, TSH: serum thyroid-stimulating hormone, FT3: serum-free triiodothyronine, FT4: serum-free thyroxine, anti-TPO Ab: anti-thyroid peroxidase antibody, Tg: thyroglobulin, anti-Tg Ab: anti-thyroglobulin antibody.

## Data Availability

All data generated or analyzed during this study are included in the published article and its supplementary information files.

## Supplementary Materials

The following supporting information can be downloaded at: https://www.mdpi.com/article/10.3390/genes13122192/s1, Table S1: The deafness-associated gene list that was used for filtration; Table S2: The list of the variants and their details after the initial filtration; Table S3: Primers for the identified variants in *SLC26A4* that were used for the co-segregation analysis by Sanger Sequencing.
