# Supplementary material for: SLC26A4 Phenotypic Variability Influences Intra- and Inter-Familial Diagnosis and Management
Source: Genes (Basel). 2022 Nov 23;13(12):2192. doi: 10.3390/genes13122192 (PMC9778369; doi:10.3390/genes13122192)
Supplement: Supplementary file 1 [file genes-13-02192-s001.zip › Table S2 SLC26A4 variants short list.docx]

| Table S2. The list of the variants and their details after the initial filtration | | | | | | | | | | | | |
| --- | --- | --- | --- | --- | --- | --- | --- | --- | --- | --- | --- | --- |
| **Family** | **Proband ID** | **Variant coordinate hg38** | **rs number** | **Mutated gene** | **Transcript** | **Exon** | **HGVS cDNA** | **HGVS**  **amino acid** | **Consequence** | **Ref/ Alt** | **Zygosity** | **Initial**  **Prediction before co-segregation analysis** |
| F1 | F1: IV-3 | chr7:107663295 |  | *SLC26A4* | NM_000441.1 |  | c.165-1G>C |  | Splice acceptor | REF: G ALT: C | Homozygous | Likely Path |
|  |  | chr2:73385909 |  | *ALMS1* | NM_015120.4 | 1/23 | c.72_74dup | p.(Glu29dup) | Inframe insertion | REF: -  ALT: GGA | Heterozygous | VUS |
|  |  | chr2:127257685 | rs779748578 | *ERCC3* | NM_000122.1 | 15/15 | c.2260G>A | p.(Asp754Asn) | Missense | REF: C  ALT: T | Heterozygous | VUS |
|  |  | chr3:53800340 | rs148898845 | *CACNA1D* | NM_000720.3 | 42/49 | c.5075G>A | p.(Arg1692Gln) | Missense | REF: G  ALT: A | Heterozygous | VUS |
|  |  | chr10:71773367 | rs146887444 | *VSIR* | NM_022153.1 | 1/7 | c.73G>A | p.(Ala25Thr) | Missense | REF: C  ALT: T | Heterozygous | VUS |
|  |  | chr11:72108851 | rs570166217 | *LRTOMT* | NM_001145308.4 | 7/7 | c.802C>T | p.(His268Tyr) | Missense | REF: C  ALT: T | Heterozygous | VUS |
|  |  | chr17:68271583 | rs144470811 | *SLC16A6* | NM_004694.4 | 5/6 | c.577A>G | p.(Ile193Val) | Missense | REF: T  ALT: C | Heterozygous | VUS |
|  |  | chr19:8522527 | rs201982814 | *MYO1F* | NM_012335.3 | 27/28 | c.3070G>A | p.(Val1024Met) | Missense | REF: C  ALT: T | Heterozygous | VUS |
|  |  | chr22:37733300 |  | *TRIOBP* | NM_001039141.2 | 8/24 | c.3950A>G | p.(Lys1317Arg) | Missense | REF: A  ALT: G | Heterozygous | VUS |
|  |  | chrX:108157191 | rs765464545 | *COL4A6* | NM_033641.3 | 45/45 | c.4882C>T | p.(Arg1628Trp) | Missense | REF: G  ALT: A | Heterozygous | VUS |
|  |  | chr12:80310633 | rs150426222 | *OTOGL* | NM_173591.3 | 29/58 | c.3329T>C | p.(Ile1110Thr) | Missense | REF: T  ALT: C | Heterozygous | Likely Benign |
|  |  | chr12:80366589 | rs149249642 | *OTOGL* | NM_173591.3 | 52/58 | c.6256A>G | p.(Ile2086Val) | Missense | REF: A  ALT: G | Heterozygous | Likely Benign |
|  |  | chr12:80370580 | rs146572555 | *OTOGL* | NM_173591.3 | 55/58 | c.6599C>T | p.(Thr2200Ile) | Missense | REF: C  ALT: T | Heterozygous | Likely Benign |
|  |  | chr12:132621866 | rs115260724 | *P2RX2* | NM_170683.3 | 10/10 | c.1388G>A | p.(Arg463Gln) | Missense | REF: G  ALT: A | Heterozygous | Likely Benign |
|  |  | chr12:132621313 | rs201474543 | *P2RX2* | NM_170683.3 | 9/10 | c.964G>A | p.(Gly322Arg) | Missense | REF: G  ALT: A | Heterozygous | Benign |
|  |  | chr16:75640355 | rs370077957 | *KARS* | NM_001130089.1 |  | c.307-7_307-6del |  | Splice region Intron | REF: AA  ALT: - | Heterozygous | Benign |
| F2 | F2:IV-1 | chr5:132312187 |  | *SLC22A4* | NM_003059.2 | 2/10 | c.420G>A | p.(Trp140Ter) | Stop gained | REF: G  ALT: A | Heterozygous | Likely Path |
|  |  | chr7:107695941 |  | *SLC26A4* | NM_000441.1 | 13/21 | c.1446G>A | p.(Trp482Ter) | Stop gained | REF: G  ALT: A | Heterozygous | Likely Path |
|  |  | chr2:73385906 |  | *ALMS1* | NM_015120.4 | 1/23 | c.69_74dup | p.(Glu28_Glu29dup) | Inframe insertion | REF: -  ALT: GGAGGA | Heterozygous | VUS |
|  |  | chr2:73386170 | rs1180446025 | *ALMS1* | NM_015120.4 | 1/23 | c.305G>A | p.(Gly102Asp) | Missense | REF: G  ALT: A | Heterozygous | VUS |
|  |  | chr5:141528920 | rs200688040 | *DIAPH1* | NM_005219.4 | 22/28 | c.2800C>T | p.(Arg934Trp) | Missense | REF: G  ALT: A | Heterozygous | VUS |
|  |  | chr7:107663435 |  | *SLC26A4* | NM_000441.1 | 3/21 | c.304G>A | p.(Gly102Arg) | Missense Splice region | REF: G  ALT: A | Heterozygous | VUS |
|  |  | chr17:74864248 | rs755725171 | *FDXR* | NM_001258012.3 | 9/12 | c.1031G>A | p.(Arg344His) | Missense | REF: C  ALT: T | Heterozygous | VUS |
|  |  | chr18:46524723 |  | *LOXHD1* | NM_144612.6 | 30/40 | c.4725G>T | p.(Arg1575Ser) | Missense | REF: C  ALT: A | Heterozygous | VUS |
|  |  | chr3:45496381 | rs202009605 | *LARS2* | NM_015340.3 |  | c.1622+8G>A |  | Splice region Intron | REF: G  ALT: A | Heterozygous | Likely Benign |
|  |  | chr22:37723190 | rs201794404 | *TRIOBP* | NM_001039141.2 | 7/24 | c.634G>A | p.(Gly212Ser) | Missense | REF: G  ALT: A | Heterozygous | Benign |
| F3 | F3:V-3 | chr7:107663295 |  | *SLC26A4* | NM_000441.1 |  | c.165-1G>C |  | Splice acceptor | REF: G  ALT: C | Homozygous | Likely Path |
|  |  | chr4:155829983 | rs770754237 | *ASIC5* | NM_017419.2 | 10/10 | c.1391T>C | p.(Ile464Thr) | Missense | REF: A  ALT: G | Heterozygous | VUS |
|  |  | chr5:90791200 | rs753503932 | *ADGRV1* | NM_032119.3 | 70/90 | c.14371G>T | p.(Ala4791Ser) | Missense | REF: G  ALT: T | Heterozygous | VUS |
|  |  | chr5:170108190 | rs371025378 | *FOXI1* | NM_012188.4 | 2/2 | c.716C>T | p.(Pro239Leu) | Missense | REF: C  ALT: T | Heterozygous | VUS |
|  |  | chr10:53857227 | rs1219799617 | *PCDH15* | NM_001142763.1 | 29/35 | c.3769G>C | p.(Val1257Leu) | Missense | REF: C  ALT: G | Heterozygous | VUS |
|  |  | chr17:34638188 | rs763116200 | *TMEM132E* | NM_001304438.1 | 9/9 | c.3181C>G | p.(Pro1061Ala) | Missense | REF: C  ALT: G | Heterozygous | VUS |
|  |  | chr19:45365077 | rs201382232 | *ERCC2* | NM_000400.3 | 6/23 | c.442C>T | p.(His148Tyr) | Missense | REF: G  ALT: A | Heterozygous | VUS |
|  |  | chr20:35476558 | rs145878385 | *CEP250* | NM_007186.5 | 16/35 | c.1826C>T | p.(Ala609Val) | Missense | REF: C  ALT: T | Heterozygous | VUS |
|  |  | chr11:121168762 | rs144343770 | *TECTA* | NM_005422.2 | 19/23 | c.5836T>C | p.(Tyr1946His) | Missense | REF: T  ALT: C | Heterozygous | Likely Benign |
|  |  | chr17:17793780 | rs371983878 | *RAI1* | NM_030665.3 | 3/6 | c.852_872del | p.(Gln285_Gln291del) | Inframe deletion | REF: CAGCAGCAGCAGCAGCAGCAG  ALT: - | Heterozygous | Likely Benign |
|  |  | chr17:50195974 | rs375914028 | *COL1A1* | NM_000088.3 | 16/51 | c.1005T>A | p.(Gly335=) | Splice region Synonymous | REF: A  ALT: T | Heterozygous | Likely Benign |

Abbreviations: HGVS, Human Genome Variation Society; Ref, reference allele; Alt, alternative allele; path; pathogenic; VUS, variant of uncertain significance
