# Supplementary material for: SLC26A4 Phenotypic Variability Influences Intra- and Inter-Familial Diagnosis and Management
Source: Genes (Basel). 2022 Nov 23;13(12):2192. doi: 10.3390/genes13122192 (PMC9778369; doi:10.3390/genes13122192)
Supplement: Supplementary file 1 [file genes-13-02192-s001.zip › Table S3 SLC26A4 primers.docx]

Table S3: Primers for the identified variants in *SLC26A4* that were used for the co-segregation analysis by Sanger sequencing

| **Variant (HGVS nomenclature)** | **Forward primer**  **5` - Oligo Seq - 3`** | **Reverse primer**  **5` - Oligo Seq - 3`** | **Amplicon size (bp)** | **annealing temperature**  **(**°C) |
| --- | --- | --- | --- | --- |
| NM_000441.1:c.304G>A | CCCAAATACCGAGTCAAGGA | CAGCAGTTTCCCAGGAAGAG | 266 | 60 |
| NM_000441.1:c.1446G>A | AGCCTGGGCAATAGAGTGTG | AACGAAAGAAAGTGGCTTCA | 356 | 60 |
| NM_000441.1:c.165-1G>C | CACTTAATCCTGTGTTCTCTAGA | GTAAGCAACCATCTGTCACAG | 452 | 60 |
